# Access to Any Site Directed Stable Isotope (^2^H, ^13^C, ^15^N, ^17^O and ^18^O) in Genetically Encoded Amino Acids

**DOI:** 10.3390/molecules18010482

**Published:** 2013-01-02

**Authors:** Prativa B. S. Dawadi, Johan Lugtenburg

**Affiliations:** Leiden Institute of Chemistry, Leiden University, P.O. Box 9502, 2300 RA, Leiden, The Netherlands; E-Mail: p.b.s.dawadi@gmail.com

**Keywords:** amino acids, isotope labelling, [5-^13^C]-leucine, [4-^13^C]-valine, ^13^C or ^15^*N*-enriched L-lysine, [^18^O]-benzylchloromethyl ether, [^13^C]-benzonitrile

## Abstract

Proteins and peptides play a preeminent role in the processes of living cells. The only way to study structure-function relationships of a protein at the atomic level without any perturbation is by using non-invasive isotope sensitive techniques with site-directed stable isotope incorporation at a predetermined amino acid residue in the protein chain. The method can be extended to study the protein chain tagged with stable isotope enriched amino acid residues at any position or combinations of positions in the system. In order to access these studies synthetic methods to prepare any possible isotopologue and isotopomer of the 22 genetically encoded amino acids have to be available. In this paper the synthetic schemes and the stable isotope enriched building blocks that are available via commercially available stable isotope enriched starting materials are described.

## 1. Introduction

**Figure 1:**
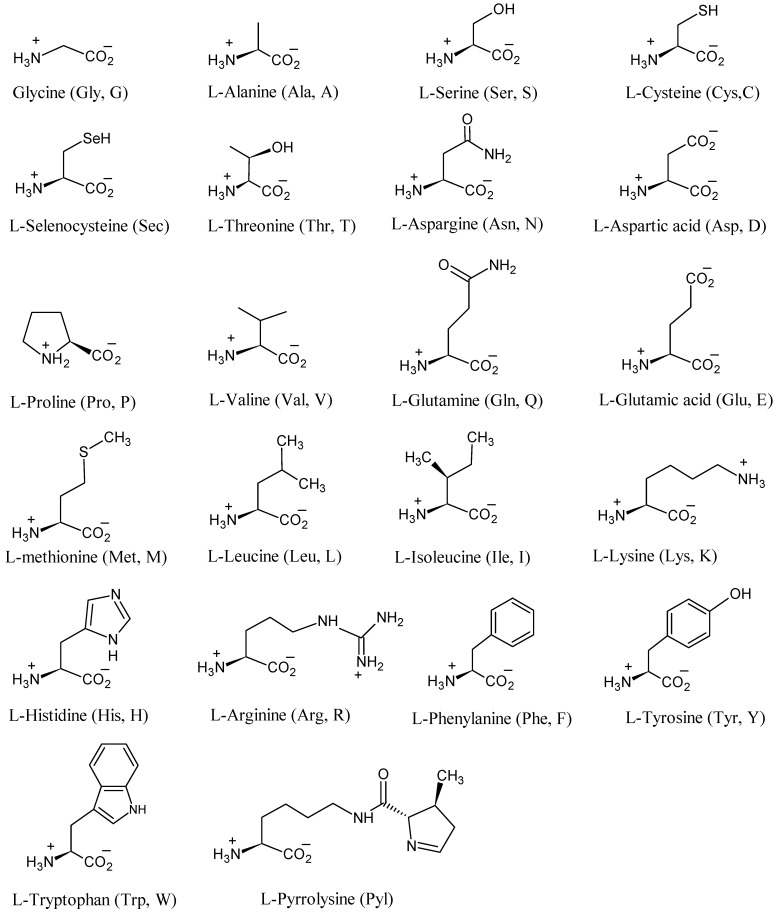
Genetically encoded amino acids (Gly (G), Ala (A), Ser (S), Cys (C), Sec, Thr (T), Asn (N), Asp (D), Pro (P), Val (V), Gln (Q), Glu (E), Met (M), Leu (L), Ile (I), Lys (K), His (H), Arg (R), Phe (F), Tyr (Y), Trp (W) and Pyl are listed based on the number of carbon atoms and amino acids with the same number of carbon atoms are listed based on increasing atomic mass.

Proteins and peptides play a preeminent role in living cells, such as receptor action, enzyme catalysis, transport and storage, hormone action, mechanical support, immune protection, *etc*. [1]. The 22 genetically encoded amino acids that are the building blocks of proteins and peptides are depicted in Figure 1 [2]. All amino acids except Gly have the L-configuration at the chiral α-carbon atom. Ile and Thr have two chiral atoms and Pyl has three chiral atoms. Tyr, Val, Met, Leu, Ile, His, Lys, Phe, Arg, and Trp are essential amino acid for mammals. These amino acids must be available in their food, as mammals are incapable of synthesizing or of synthesizing them in sufficient amount to meet metabolic needs.

After the primary protein systems are formed via translation of DNA and RNA, post-translational modifications of residues that include acylation, phosphorylation, sulfation, hydroxylation, oxidative methylation, prenylation and cross-linking occur that lead to the active proteins and peptides in the living cell. In order to understand the role of proteins for the structure-function, dynamics and localization of the individual proteins in the complex environment of living cells, techniques such as labeling endogenous proteins have been applied [3]. A drawback of these labeling methods is that modifications are introduced on the protein systems in question. Site-directed stable isotope introduction on the other hand, allows labeling in the protein system without any modification.

Nowadays, ^13^C-, ^15^N-enriched proteins are detected in prokaryotic cells using NMR techniques [4]. Earlier, methyl groups were used for the NMR detection of proteins in cells [5]. Stable isotope enriched systems have been used to study protein structure with 2D-Heteronuclear NMR techniques [6]. Somewhat later, methods for preparing either U-^13^C or U-^15^N or both U-^13^C and U-^15^N with 99% stable isotope incorporation in the genetically encoded amino acids became available. This has led to a quantum jump in the application of NMR techniques in the study of proteins. The NMR methods have been optimized by using U-^13^C, U-^15^N-amino acids in which only one proton is stereoselectively replaced by deuterium at a methylene group (stereo-array isotope labeling—SAIL) [7,8]. Many more important new NMR techniques for the study of proteins have been reported [9,10,11,12,13,14]. In addition to the NMR methods, IR techniques are also used to study the protein function [15]. In general the required isotopically labeled proteins are prepared by using genetic expression techniques; one of the optimal methods of this technique is the use of cell-free synthesis [16]. The preparation of proteins with isotopically enriched amino acids via genetic techniques has as a main drawback that all amino acid residues of one type in the protein chain will be enriched.

At the moment chemical total synthesis is an active and a fruitful research field that allows the site-directed stable isotope incorporation at any specific amino acid residue in a protein molecule [17]. 

The first native chemical ligation procedure has been developed based on the cystein residues. Later, a method was developed to make a new peptide bond via deselenization of piptidyl selenoester where sulfur atom from corresponding thioester of cystein residue is replaced with selenium atom [18,19,20]. Native chemical ligation of hydrophobic peptides that are insoluble in water has also been revealed [21]. A general method of chemoselective ligation involves decarboxylative condensation of an α-keto acid of a peptide and a hydroxyl amine function of another peptide to make a new amide bond of the expected peptide [22].

In order to study the site-directed stable isotopically labeled proteins with the known and newly developed isotope sensitive non-invasive techniques, the access to any stable isotopologue and stable isomer of the genetically introduced amino acids is essential. In this paper the synthetic and chemoenzymatic methods to get access to these systems will be discussed.

Known L-α-amino acids labeled with stable isotopes at specific positions have been reported [23,24]. Synthetic methods are optimized to resolve problems due to diastereotopic methyl groups, hydrogen atoms and additional chiral centers. The synthetic schemes discussed in this paper are easily simplified when these problems are not present for the isotopomer in question. The use of possible isotopologues of amino acids in a protein molecule will allow the use of mass spectral techniques that plays an important role in the field of metabolomics and proteomics [25,26,27]. In addition, the possibility of introducing ^18^O isotopes in amino acid residues has aided the use of mass spectrometry in the protein study [28].

## 2. Synthetic Schemes

A number of simple highly stable isotope (^2^H, ^13^C, and ^15^N), ^17^O (70%) and ^18^O (97%) enriched building blocks are commercially available. Except glycine all amino acids in Figure 1 exist in the L-form. This gives a (2*S*)-configuration except in Cys and Sec. These amino acids have a (2*R*)-configuration due to the presence of S and Se atoms, respectively. Ile and Thr have (2*S*, 3*S*)- and (2*S*, 3*R*)-configurations, respectively.

The synthetic schemes should start from achiral building blocks wherein high enantioselectivity is achieved by using chiral catalysts, chiral phase transfer catalysts and enzymes. The use of chiral templates that require additional chemical reaction steps should be avoided. The synthetic schemes should result in the product without isotopic loss, dilution or scrambling. The schemes should give well-defined synthetic methods in the case of the presence of diasteretopic methyl groups (Val, Leu) or diasteretopic hydrogen atoms (except Ala, Val and Thr).

The schemes discussed in this paper are optimized to meet these requirements for the synthesis of all possible isotopologues and isotopomers. General methods (Scheme 1,Scheme 2,Scheme 3,Scheme 4,Scheme 5,Scheme 6) are indicated in the specific Roman numbers whereas the synthesis of 22 amino acids (Schemes 7,8,9,10,11,12,13,14,15,16,17,18,19,20,21,22,23,24,25,26,27,28,29,30) are indicated in Arabic numbers. When building blocks from the general schemes are used in the specific schemes they maintain their Roman numbers. Based on the required isotope enrichment in the system, many synthetic schemes can easily be simplified. 

## 3. General Methods to Synthesize L-Amino Acids

### 3.1. Catalytic Reduction of 2,3-Dehydroamino Acids I

**Scheme 1:**
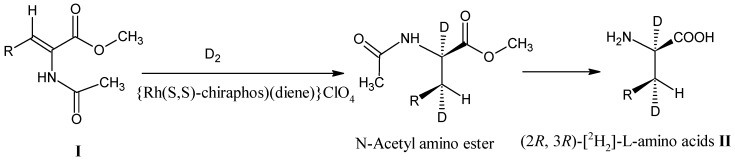
*N*-Acetyl-2,3-dehydroamino acid derivatives **I** occur in the thermodynamically stable *Z*-form. Catalytic reduction with D_2_ in the presence of chiral catalyst and subsequent hydrolysis gives (2*R*, 3*R*)-[^2^H_2_]-L-amino acids **II**.

*N*-Acetyl-2,3-dehydroamino acid esters **I** are easily available via elimination reactions of β-substituted *N*-acetyl amino acid esters [29,30,31]. Asymmetric hydrogenation of 2,3-dehydroamino acid esters gives an access to prepare a wide range of amino acids. *N*-Acetyl-2,3-dehydroamino acid derivatives **I** have been treated with D_2_ (^2^H_2_) in the presence of a chiral Rh catalyst to afford (2*R*, 3*R*)-[^2^H_2_]-L-amino acids **II** (Scheme 1) [32]. Using the [3-^2^H]-2,3-dehydroamino acid derivatives it is possible to obtain (3*S*)-[3-^2^H]-L-amino acids in highly enantiomeric pure form [33,34].

### 3.2. Reductive Amination of α-Keto Acids **IV**

**Scheme 2:**
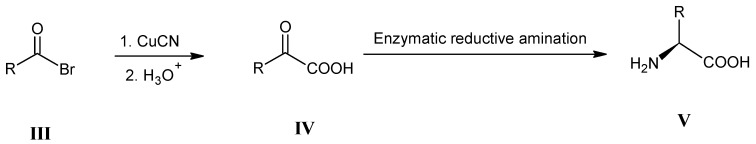
Enzymatic reductive amination of α-keto acids **IV** to afford [^2^H, ^15^N or ^13^C]-amino acids **V**.

α-Keto acids **IV** of the corresponding amino acids are easily available from acid bromides **III**. Treatment of acid bromides **III** with copper (I) cyanide and subsequent hydrolysis followed by enzymatic reductive amination gives the corresponding enantiomeric pure amino acids **V** [35,36]. The synthetic method described in the Scheme 2 allows to the incorporation of [2-^2^H]-, [2-^15^N]- and [^13^C]-isotopes in the corresponding amino acids **V**. 

### 3.3. Hydrolysis of α-Amino Nitriles **VII**

**Scheme 3:**
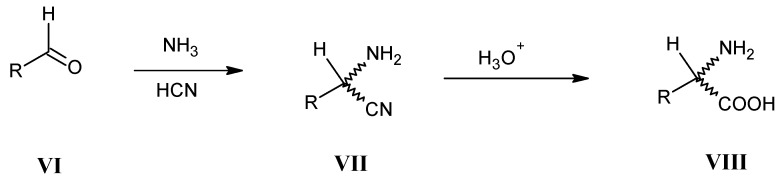
Preparation of D,L-mixtures of α-amino nitriles **VII** via the Strecker reaction.

In the Strecker reaction aldehydes **VI** are treated with ammonia in the presence of hydrocyanic acid to give D,L-mixtures of α-amino nitriles **VII** followed by hydrolysis to afford D,L-mixtures of amino acids **VIII** (Scheme 3) [37,38].

**Scheme 4:**
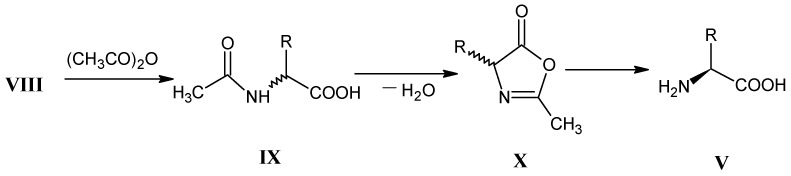
Preparation of enantiomeric pure L-amino acids **V** via dynamic kinetic resolution of oxazol-5-(4*H*)-ones **X**.

Treatment of the D,L-mixture of α-amino nitrile **VII** with an enzyme nitrilase gives a separable mixture of L-α-amino acid and D-α-amino nitrile. In general the presence of a chiral catalyst does not lead to an enantiomeric pure form except for the synthesis of valine [39]. In Scheme 4 it is shown that D,L-α-amino acid **VIII** can be converted into the oxazol-5-(4*H*)-ones **X** (azlactones) via *N*-acetylated glycine ester derivative **IX**. Following the dynamic kinetic resolution procedure the corresponding L-α-amino acid derivative **V** can be separated from D,L-α-amino acids **VIII** [40,41].

**Scheme 5:**
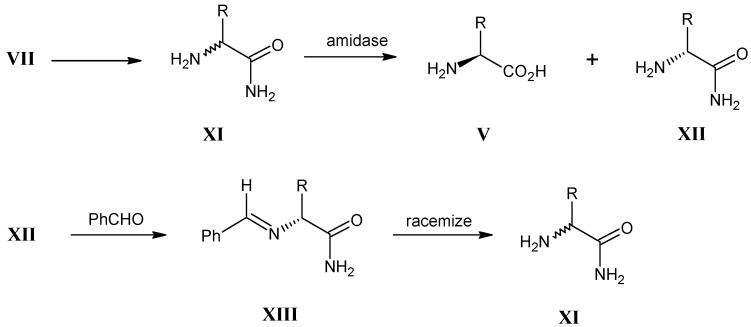
The conversion of D,L-α-amino nitriles **VII** into D,L-α-amino amides **XI** to afford L-α-amino acids **V**.

Another approach is shown in Scheme 5 for the conversion of D, L-α-amino nitriles **VII** into the corresponding D,L-α-amides **XI**. The final hydrolysis of the mixture with an enzyme amidase gives pure L-α-amino acids **V** and pure D-α-amino amides **XII**. D-α-Amino amides **XII** are simply racemized via an intermediate benzalimine to form a second batch of D, L-α-amino amides **XI** [36].

### 3.4. Alkylation of N-(Diphenylmethylene)glycine tert-Butyl Ester XIV (O’Donnell Method)

**Scheme 6:**
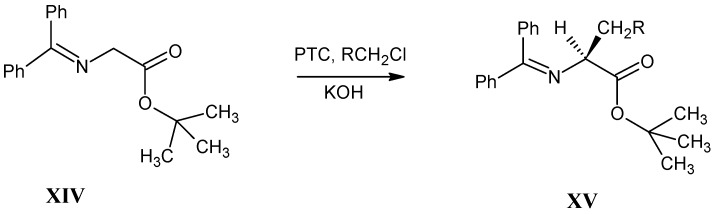
Monoalkylation of *N*-(diphenylmethylene)glycine *tert*-butyl ester **XIV** with phase transfer catalyst *O*-allyl-*N*-(9-anthracenylmethyl)cinchonidinium bromide with primary halides to give the N-protected L-α-amino acid derivatives **XV**.

In Scheme 6 it is shown that the *N*-(diphenylmethylene)glycine *tert*-butyl ester **XIV** is converted into protected monoalkylated L-α-amino acid derivative **XV** in a high yield with a high enantiomeric excess. These derivatives are easily converted into the corresponding amino acids [42,43,44,45].

## 4. Synthesis of 22 Amino Acids

### 4.1. Glycine

Glycine serves as a building block of peptides and proteins. Stable isotope enriched glycine derivatives function as the starting materials to introduce stable isotopes (^2^H, ^13^C and ^15^N) at the α-carbon, the carboxylic acid and the amino group of all L-α-amino acids. 

**Scheme 7:**
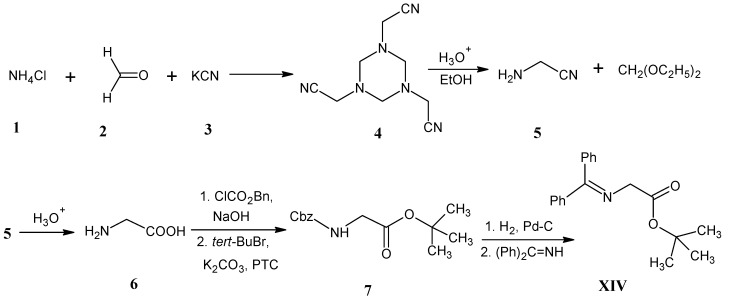
Synthesis of 2-aminoacetonitrile (**5**) and its conversion into *N*-(diphenylmethylene)-glycine *tert*-butyl ester **XIV** for the preparation of glycine in all possible isotope enriched forms.

In Scheme 7 it is indicated that starting materials NH_4_Cl, HCHO and KCN are used for the preparation of glycine and *N*-(diphenylmethylene)glycine *tert*-butyl ester **XIV**. The Strecker reaction of NH_4_Cl (**1**), two equivalents of HCHO (**2**) and KCN (**3**) afforded the product hexahydro-1,3,5-triazine-1,3,5-tris(acetonitrile) (**4**) [46]. Treatment of compound **4** with acid in ethanol yielded 2-aminoacetonitrile (**5**) and diethoxymethane. Subsequent acid hydrolysis of the compound **5** gave glycine (**6**). Starting materials NH_4_Cl (**1**), HCHO (**2**) and KCN (**3**) are commercially available in all possible stable isotope enriched forms.

The amino and carboxyl groups of glycine (**6**) are protected using benzyl chloroformate and *tert*-butyl bromide, respectively, to afford *N*-protected glycine *tert*-butyl ester **7** [47]. Hydrogenation of *N*-benzyloxycarbonyl glycine *tert*-butyl ester (**7**) with palladium on charcoal yielded the *tert*-butyl ester of glycine with a free amino group. Reaction with commercially available 1,1-diphenylmethyl-eneimine afforded *N*-(diphenylmethylene)glycine *tert*-butyl ester **XIV** [47].

**Scheme 8:**
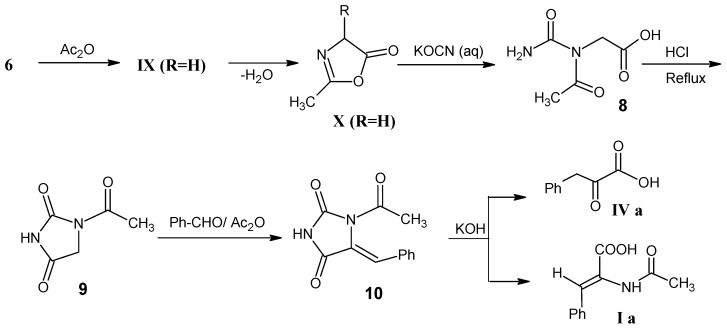
The conversion of glycine (**6**) into oxazol-5-(4*H*)-one X (R = H) and 1-acetylimidazolidine-2,4-dione (**9**).

The synthetic methods depicted in Scheme 8 show that glycine (**6**) can be converted into oxazol-5-(4*H*)-one **X** (R = H) via N-acetyl glycine **IX** (R = H). Treatment of compound **X** (R = H) with potassium cyanate afforded carbamide **8**, that is refluxed in aqueous HCl to obtain 1-acetylimidazolidine-2,4-dione (**9**) [48]. Hydantoin derivative **9** is treated with benzaldehyde via a Knoevenagel reaction followed by hydrolysis of product **10** to afford the corresponding α-keto acid **IVa** or *N*-acetyl-2,3-dehydroamino acid derivative **Ia** [49,50].

**Scheme 9:**
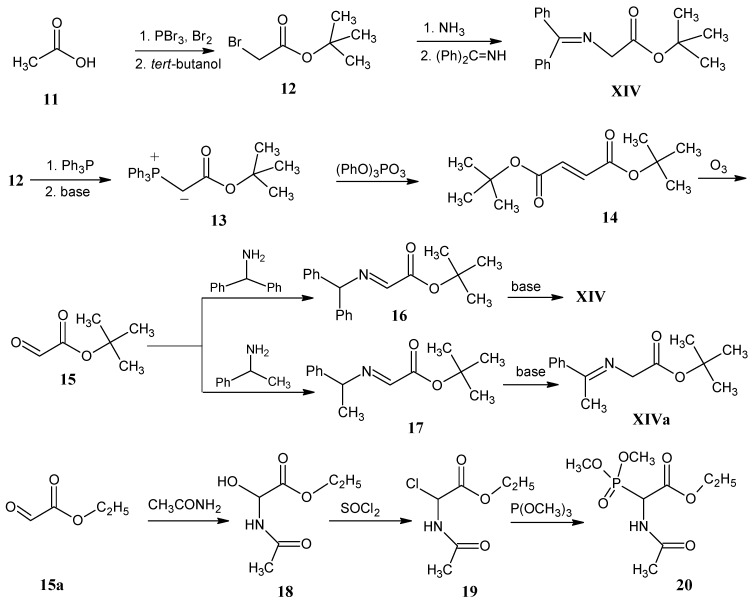
The conversion of acetic acid (**11**) into *N*-(diphenylmethylene)glycine *tert*-butyl ester **XIV** and *N*-(methylphenylmethylene)glycine *tert*-butyl ester **XIVa** via *tert*-butyl glyoxalate (**15**). Conversion of ethyl glyoxalate (**15a**) into N-acetyl phosphonato glycine ethyl ester (**20**). Products **XIV**, **XIVa**, **15** and **20** can be obtained in any ^13^C and ^15^N position to enrich glycine part with stable isotopes.

Scheme 9 illustrates the conversion of acetic acid (**11**) into 2-bromoacetic acid via a Hell-Volhard-Zellinsky reaction. Esterification of 2-bromoacetic acid with *tert*-butanol afforded *tert*-butyl 2-bromoacetate (**12**) [47]. Treatment of ester **12** with NH_3_ yielded the *tert*-butyl ester of glycine (**6**) [51]. Transimination of *tert*-butyl ester of glycine (**6**) with benzophenoneimine afforded *N*-(diphenylmethylene)glycine *tert*-butyl ester **XIV**. 

*tert*-Butyl 2-bromoacetate (**12**) with triphenylphosphine afforded the corresponding phosphonium bromide that in the presence of base gave ylide **13**. One equivalent of compound **13** with 0.5 equivalents of triphenyl phosphite ozonide (TPPO) yielded di-*tert*-butyl fumarate (**14**). *tert*-Butyl glyoxalate (**15**) is obtained via ozonolysis of ester **14** [52]. Imine **16** is obtained by the reaction of *tert*-butyl glyoxalate (**15**) with diphenylmethylamine. Upon base treatment of imine **16 ***N*-(diphenylmethylene)glycine *tert*-butyl ester **XIV** is obtained that served as the starting material for the O’Donnell alkylation [53]. Similarly, reaction of glyoxalate **15** with racemic 1-phenylethylamine (prepared by reductive amination of acetophenone with ammonia) afforded the imine **17** which upon base treatment is converted into *N*-(methylphenylmethylene)glycine *tert*-butyl ester **XIVa**. 

Ethyl glyoxalate (**15a**) with acetamide afforded *N*-acetyl 2-hydroxyglycine *tert*-butyl ester (**18**). Upon treatment with SOCl_2_, the hydroxyl group is substituted by a chlorine atom to afford compound **19**. An Arbuzov reaction with trimethyl phosphite yielded the ethyl ester of *N*-acetyl-2-dimethyl phosphonato glycine (**20**). 2,3-Dehydroamino acid derivatives **I** can be achieved via Wittig reactions of product **20** with appropriate aldehydes [54,55].

### 4.2. Alanine

**Scheme 10:**
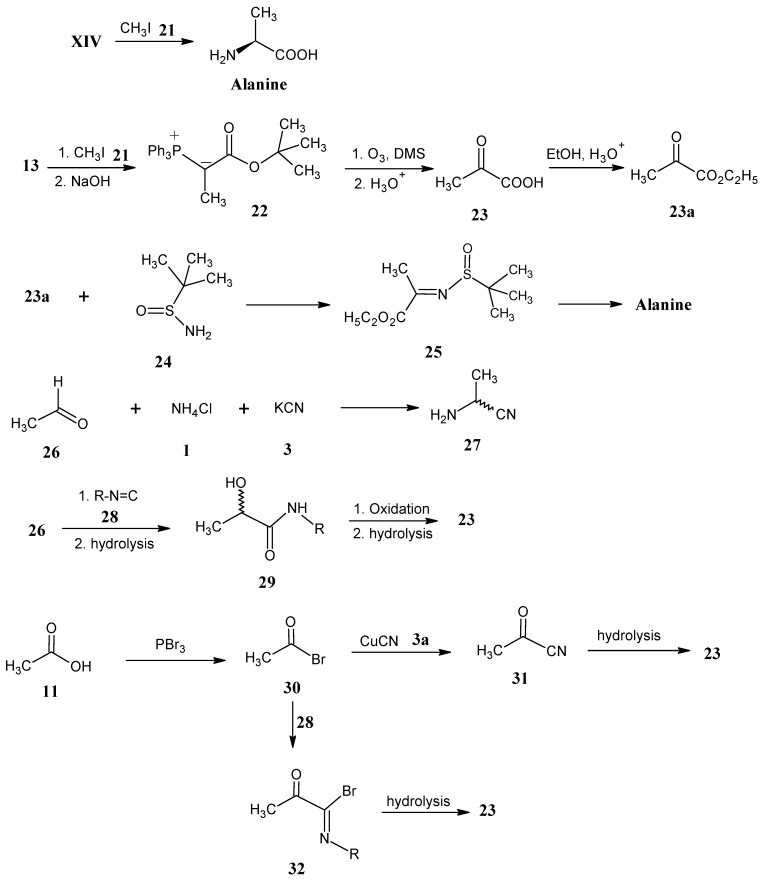
The preparation of isotopically enriched L-alanine in all possible positions with isotopically labeled commercially available building blocks.

In Scheme 10 it is indicated that treatment of CH_3_I (**21**) under O’Donnell conditions with protected glycine gave a high yield of L-α-alanine [56].

Reaction of CH_3_I (**21**) with *tert*-butyl 2-(triphenylphosphonium)acetate (**13**) in the presence of a base afforded the ylide *tert*-butyl 2-(triphenylphosphonium)propionate (**22**, Scheme 10). Pyruvic acid (**23**) is obtained by the ozonolysis of the ylide **22**. Esterification of pyruvic acid in ethanol afforded ethyl pyruvate (**23a**). Reaction of ethyl pyruvate (**23a**) with 2-methylpropane-2-sulfinamide (**24**) afforded imine **25** that upon reaction with L-Selectride resulted in L-alanine ethyl ester. L-Alanine is obtained after saponification in 98% yield [57].

The Strecker reaction of acetaldehyde (**26**) with NH_4_Cl (**1**) and KCN (**3**) yielded racemic 2-aminopropionitrile (**27**). Earlier, the conversion of 2-aminopropionitrile (**27**) into L-alanine has been discussed (Scheme 3). Furthermore, reaction of acetaldehyde (**26**) with isocyanides **28** followed by hydrolysis formed racemic α-hydroxy amides **29** [58]. Subsequent oxidation with KMnO_4_ and hydrolysis afforded pyruvic acid (**23**) [59].

Acetyl bromide (**30**) is obtained by the reaction of acetic acid (**11**) with PBr_3_ and subsequent treatment with CuCN (**3a**) gave pyruvic nitrile **31**. Hydrolysis of nitrile **31** afforded pyruvic acid (**23**) in 50% yield [60,61]. Acetyl bromide (**30**) is reacted with isocyanides **28** to obtain imine derivative **32**. Hydrolysis of the imine compound **32** afforded pyruvic acid (**23**). The preparation of ^13^C-labeled pyruvic acid (**23**) at any position and combinations of positions is possible via ^13^C-labeled isocyanides **28** [62,63]. ^13^C-Labeled isocyanides **28** are easily accessible via dehydration of formylamines. The conversion of pyruvic acid (**23**) into L-alanine has been discussed before (Scheme 2) [64,65,66]. The isotopic enrichment of all atoms that constitute L-alanine is accessible via the availability of highly isotope-enriched building blocks. 

### 4.3. Serine

**Scheme 11:**
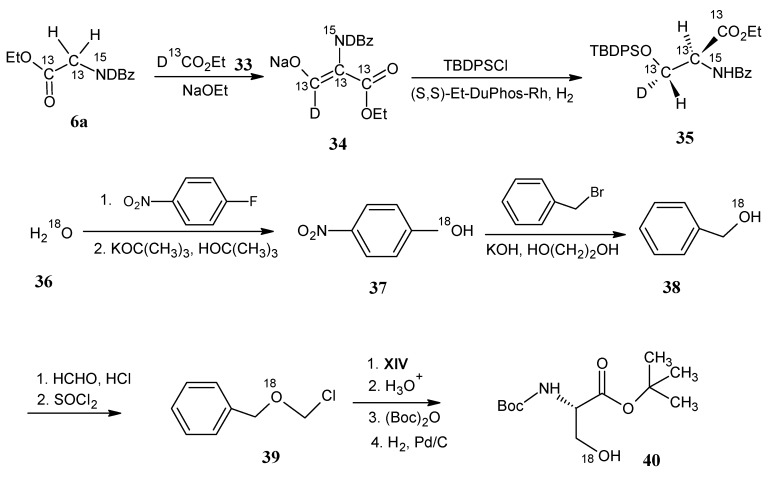
The preparation of isotopically enriched serine.

In Scheme 11 it is indicated that stable isotope enriched *N*-benzoylglycine ethyl ester **6a** is treated with stable isotope enriched formate **33** to form the 2,3-didehydroderivative **34**. Compound **34** is converted into the corresponding *tert*-butyldiphenyl silyl ether that is subsequently hydrogenated with a chiral rhodium catalyst to give stable isotope enriched serine derivative **35** [67]. 

In order to enrich the hydroxyl group with ^18^O (and ^17^O) isotope the reactions in the lower part of Scheme 11 are carried out. 4-Nitrofluorobenzene is treated with H_2_^18^O (**36**) in *tert*-butanol with one equivalent potassium *tert*-butanolate to obtain [^18^O]-4-nitrophenolate **37**. This phenolate is treated with benzyl bromide to give the [^18^O]-benzyl-4-nitrophenylether which upon treatment with KOH in ethylene glycol gave [^18^O]-benzyl alcohol **38** without any ^18^O loss or dilution. Treatment of the alcohol **38** with formaldehyde in the presence of HCl gave (benzyloxy)methanol that is treated with thionyl chloride to obtain [^18^O]-benzylchloromethyl ether **39** [68]. The *N*-protected glycine ester **XIV** is treated with compound **39** in the presence of a base and phase transfer catalyst. The *N*-protecting benzophenoneimine group is removed by treatment with citric acid followed by a reaction using Boc-anhydride and triethylamine to afford *N*-Boc, *O*-benzyl serine *tert*-butyl ester. Catalytic hydrogenation of this compound yielded serine derivative **40** with a free hydroxyl group. Final deprotection of the amino group and ester hydrolysis afforded L-serine which is now accessible in any stable isotope-enriched form using commercially available building blocks [69].

### 4.4. Cysteine and Selenocysteine

**Scheme 12:**
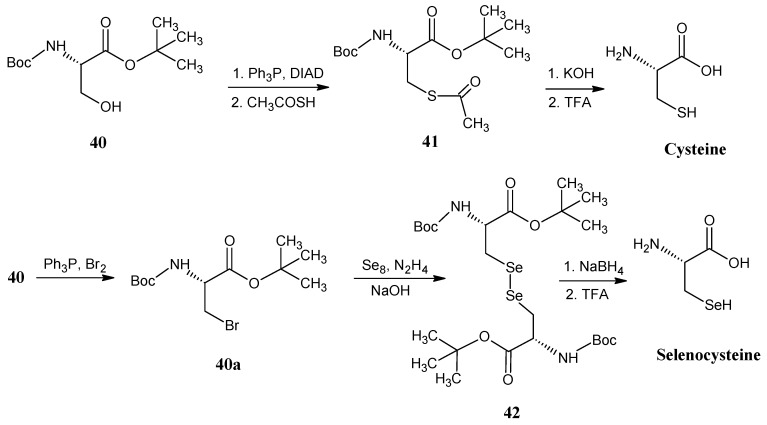
Preparation of cysteine and selenocysteine starting from *N*-protected serine **40**.

In Scheme 12 it is indicated that the protected serine derivative **40** is converted via a Mitsonobu reaction into *N*-protected *S*-acetyl cysteine **41** that after base induced deacetylation and acid catalyzed *N*-deprotection afforded cysteine [69].

Treatment of *N*-protected serine derivative **40** with bromine and triphenylphosphine in the presence of imidazole afforded *N*-protected 3-bromo serine **40a**. Reaction of *N*-protected 3-bromo serine **40a** with Se_8_ and hydrazine in the presence of NaOH yielded the diselenide derivative **42**. After sodium borohydride reduction and acid catalyzed amino group deprotection selenocysteine is obtained [69]. Besides selenocysteine, upon catalytic reduction of *N*-protected 3-bromo serine derivative **40a** stable isotope enriched alanine can be obtained [67]. 

### 4.5. Threonine

**Scheme 13:**
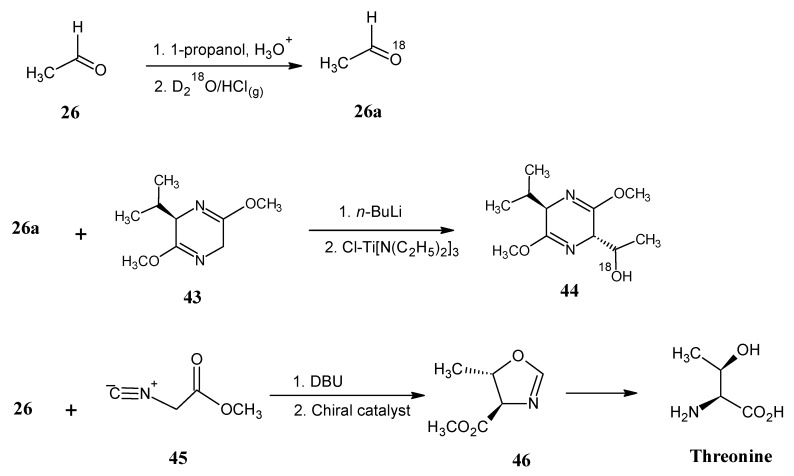
Preparation of stable isotope enriched L-threonine via the Schöllkopf method or stereo-induced oxazoline method.

Acetaldehyde (**26**) is converted into 1,1-dipropoxyethane by acid catalyzed reaction with 1-propanol. 1,1-Dipropoxyethane is mixed with D_2_^18^O in the presence of HCl_(g)_ to afford ^18^*O*-acetaldehyde (**26a**) (Scheme 13). 

Next, the bislactimether of cyclo-(D-Val-Gly) is treated with *n*-BuLi at −78 °C in THF to obtain an anion of compound **43** that is treated with chlorotitaniumtris(diethylamide). To this mixture isotopically labeled acetaldehyde (**26a**) is added to afford the required bislactimether of cyclo-(D-Val-Thr) **44** via Schöllkopf method. Hydrolysis of the product **44 **afforded methyl esters of D-valine and L-threonine. Removal of valine by cationic exchange chromatography and hydrolysis of the remaining product gave L-threonine and L-*allo*-threonine in a 15:1 ratio. These two compounds could be separated easily. It is gratifying that this method led to an optimal formation of the two chiral centers in one step [68].

Oxazolines **46** can be prepared via the reaction of aldehydes with methyl α-isocyanoacetate (**45**) in the presence of a chiral catalyst. Recently, a general method to prepare stereo-induced oxazolines **46** has been used to synthesize threonine [70].

### 4.6. Asparagine and Aspartic Acid

**Scheme 14:**
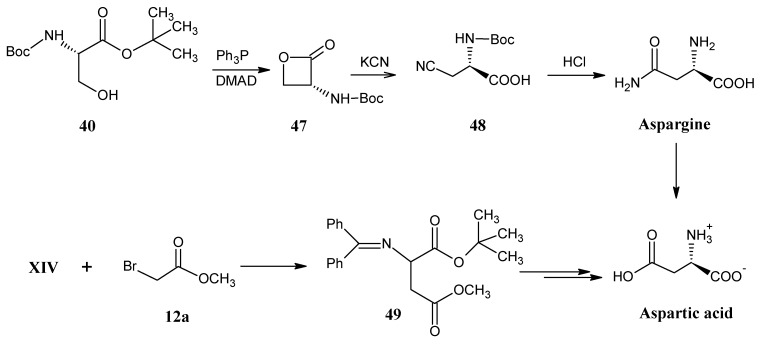
Preparation of aspargine and aspartic acid via the *N*-Boc serine *tert*-butyl ester **40** and O’Donnell method, respectively.

In Scheme 14 it is indicated that *N*-Boc serine *tert*-butyl ester (**40**) reacted with triphenylphosphine and DMAD (dimethyl azodicarboxylate) to form the β-lactone **47**. β-Lactone **47** is treated with KCN (**3**) to achieve 2-[(*tert*-butoxycarbonyl)amino]-3-cyanopropanoic acid (**48**) that upon acid catalyzed hydrolysis and deprotection afforded aspargine. Aspartic acid is obtained from aspargine after acid catalyzed hydrolysis of the amide group [71].

O’Donnell coupling between the *N*-protected glycine *tert*-butyl ester **XIV** and methyl bromoacetate (**12a**) resulted in *N*-(diphenylmethylene)aspartic acid 1-*tert*-butyl-4-methyl ester (**49**) that upon deprotection followed by saponification afforded aspartic acid [43]. An enzymatic method has been described to convert di-*tert*-butyl fumarate (**14**, symmetrically enriched with stable isotope) into aspartic acid by treatment with ammonia in the presence of immobilized enzyme aspartase [72]. 

### 4.7. Proline

**Scheme 15:**
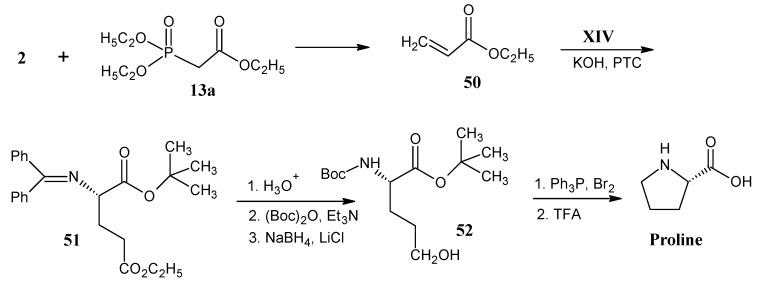
Conversion of N-(diphenylmethylene)glutamic acid 1-*tert*-butyl-5-ethyl ester (**51**) into stable isotope labeled proline.

In Scheme 15 it is depicted that reaction of HCHO (**2**) and ethyl 2-diethylphosphonoacetate (**13a**) afforded ethyl acrylate (**50**) [73].

O’Donnell reaction of ethyl acrylate (**50**) with the *N*-(diphenylmethylene)glycine *tert*-butyl **XIV** afforded the *N*-(diphenylmethylene)glutamic acid 1-*tert*-butyl-5-ethyl ester (**51**). Removal of benzophenoneimine group, Boc protection of the amino group and subsequent NaBH_4_/LiCl reduction of the ethyl ester function yielded the alcohol derivative **52**. Conversion of the primary alcohol function into the corresponding bromide is carried out with a mixture of triphenylphosphine and bromine in dichloromethane. The internal nucleophilic substitution of the free amino group led to ring closure to afford proline *tert*-butyl ester. Removal of the *tert*-butyl ester by hydrolysis with 10% TFA afforded L-proline [74].

### 4.8. Valine

**Scheme 16:**
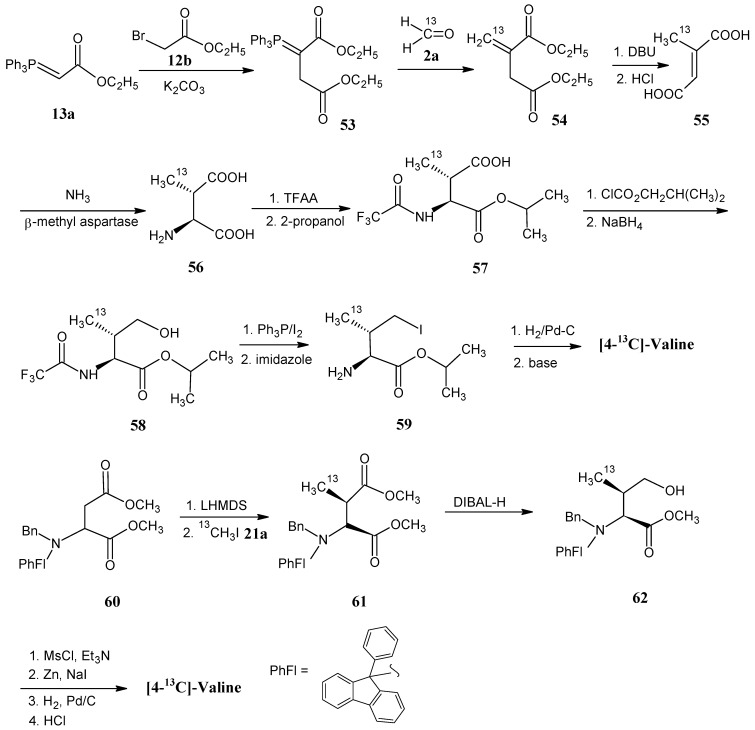
Synthesis of the carbon skeleton of valine from 2-methyl fumaric acid (**55**).

In Scheme 16 it is shown that the phosphorane **53** is obtained by the alkylation of ethyl-(triphenylphosphoranylidene)acetate (**13a**) with ethyl 2-bromoacetate (**12b**) in the presence of solid K_2_CO_3_. The phosphorane **53** is treated with H_2_C^13^O (**2a**) to otain itaconic diester **54** via the Wittig reaction. Upon treatment with DBU and heating in the presence of concentrated HCl isomerization of the exo-double bond and hydrolysis of the ester bond are effected to give pure 2-methyl fumaric acid **55**. Reaction of the product **55** with NH_3_ and β-methyl aspartase afforded 3-methyl aspartic acid (**56**). The formation of the *N*-trifluoroacetamide of the succinic acid anhydride is achieved by the addition of trifluoroacetic anhydride in THF. The ring opening of the anhydride with 2-propanol afforded the product **57 **with ester function at C-1 position. The mixed anhydride derivative of product **57** is reduced with NaBH_4_ to afford the alcohol **58**. Conversion of the primary alcohol function into the iodo compound **59** is effected by the treatment with triphenyl phosphite and iodine. The iodo function is removed by catalytic reduction and deprotection in the presence of a base to yield valine.

The ^13^C-isotope enriched [4-^13^C]-valine is prepared to show that these synthetic methods allow the chiral discrimination between the two diastereotopic methyl groups. Using ^13^C-formaldehyde (**2a**) (2*S*,3*S*)-[4-^13^C]-valine with a trace of the other enantiomeric form is obtained [47].

On the lower line in Scheme 16 it is depicted that dimethyl *N*-benzyl-*N*-(9-phenyl-9*H*-fluoren-9-yl)-aspartate (**60**) reacted with lithium hexamethyldisilazide (LHMDS) to obtain the anion of the product **60**, that subsequently underwent alkylation with ^13^C-methyl iodide (**21a**) to afford the *N*-protected ^13^C-methyl aspartic acid ester **61**. Reaction of the product **61** with DIBAL-H afforded the alcohol **62**, further protection of the hydroxyl group with mesityl chloride followed by iodine substitution, reduction and deprotection of the α-carboxylic acid ester resulted in (2*S*,3*S*)-[4-^13^C]-valine [75].

**Scheme 17:**
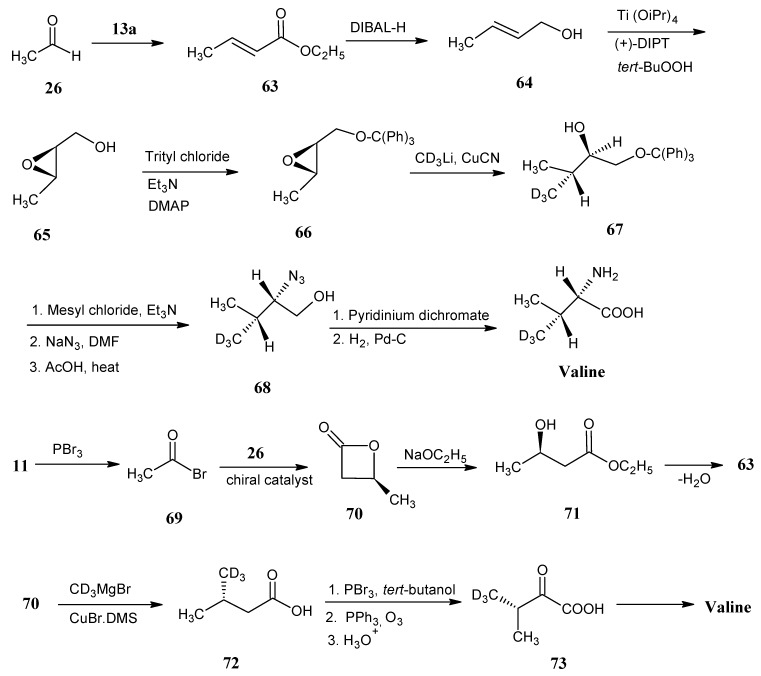
Synthesis of isotopically enriched valine starting from acetaldehyde **26** or acetic acid **11**.

In Scheme 17 it is indicated that the Wittig-Horner reaction of ethyl 2-(diethylphosphono)acetate [**13a**, prepared by the reaction of ethyl 2-bromoacetate (**12b)** and triethyl phosphite] with acetaldehyde (**26**) afforded ethyl crotonate (**63**) followed by DIBAL-H reduction to afford crotyl alcohol (**64**). These compounds are accessible in all possible stable isotopologues and isotopomers [76].

Sharpless asymmetric epoxidation of alcohol **64** gave the epoxide **65**. The epoxide is treated with (Ph)_3_C-Cl (trityl chloride) to protect the primary alcohol group as trityl ether **66**. The S_N_2 reaction with trideuteromethyl-lithium copper complex gave the enantiomeric pure deuterated derivative **67**. Mesitylation followed by reaction with the sodium azide afforded an azido derivative with trityl ether. The *O*-protection is removed by refluxing in acetic acid to obtain the azido alcohol derivative **68**. Reduction of the azide function yielded (2*S*, 3*S*)-[4-CD_3_]-valine [77].

Another simple approach to enrich isotopes in valine is via the preparation of intermediate molecule (3*S*)-3-methyl-β-butyrolactone (**70**). β-Butyrolactone **70 **is obtained by the reaction of acetyl bromide (**69**) [prepared from Hell-Volhardt-Zellinsky reaction of acetic acid (**11**) with PBr_3_] with acetaldehyde (**26**) in the presence of a base and a chiral catalyst. The ring-opening of β-butyrolactone **70** with ethoxide anion resulted in the formation of (3*S*)-3-hydrobutyrate (**71**) which could be dehydrated to obtain ethyl crotonate (**63**). Reaction of β-butyrolactone **70** with the Grignard copper complex of CD_3_I resulted in optically pure (3*R*)-3-(trideuteromethyl)butyric acid (**72**) [78].

Another alternative method of preparation of an α-keto acid **73** would be a Hell-Volhardt-Zellinsky reaction of the product **72** with PBr_3_. The corresponding bromide could be further reacted with triphenylphosphine to obtain the ylide followed by ozonolysis to afford the α-keto acid **73**. Reductive amination of α-keto acid **73** affords valine (Scheme 2). 

### 4.9. Glutamine and Glutamic Acid

**Scheme 18:**
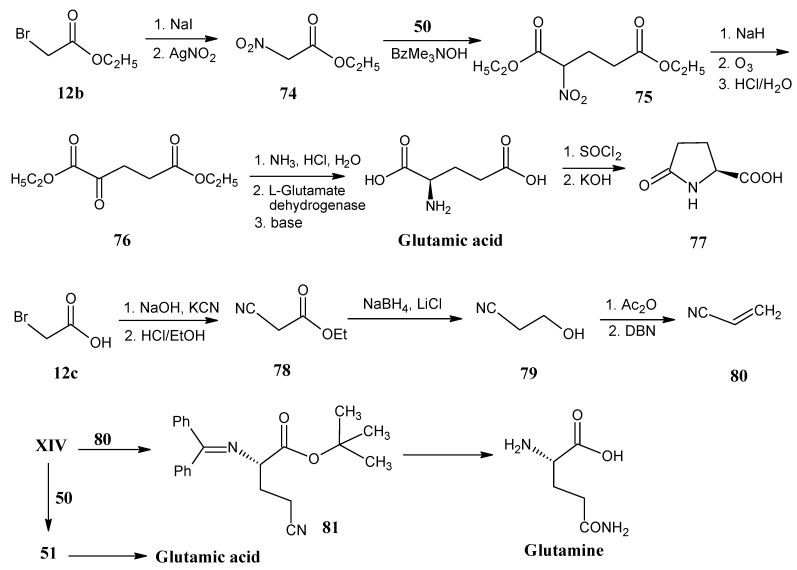
Preparation of stable isotope enriched glutamic acid and glutamine from ethyl 2-bromoacetate **12b** and 2-bromoacetic acid **12c**, respectively.

In Scheme 18 it is indicated that ethyl 2-nitroacetate (**74**) is prepared by the reaction of ethyl bromoacetate (**12b**) with NaI and AgNO_2_. Michael addition of compound **74** with ethyl acrylate (**50**) in the presence of benzyltrimethyl ammonium hydroxide afforded diethyl 2-nitroglutarate (**75**). The anion of product **75** is ozonolyzed to obtain diethyl 2-oxoglutarate (**76**). The reductive amination with ammonia in the presence of L-glutarate dehydrogenase followed by base catalyzed saponification afforded glutamic acid [73]. Glutamic acid can easily be converted into pyroglutamic acid (5-oxoproline, **77**) that offers an alternative building block for the synthesis of isotopically enriched proline [79,80].

The reaction of acrylonitrile (**80**) with the *N*-protected glycine *tert*-butyl ester **XIV** under O’Donnell conditions afforded the expected product **81**. *N*-Deprotection followed by the conversion of the nitrile function of product **81** into amide afforded glutamine [74]. A similar reaction of ethyl acrylate (**50**) with **XIV** yielded the *N*-protected ester derivative **51**. *N*-Deprotection followed by hydrolysis of the ester function of product **51** into carboxylic acid afforded glutamic acid [74]. 

All possible isotopomers of acrylonitrile (**80**) are accessible from bromoacetic acid (**12c**). Cyanoacetic acid is obtained by the reaction of bromoacetic acid (**12c**) with KCN (**3**). Esterification of the carboxylic group with ethanol afforded ethyl cyanoacetate (**78**) followed by the reduction with NaBH_4_ in the presence of LiCl to obtain 2-cyanoethanol (**79**). Acrylonitrile (**80**) is obtained by treatment of the alcohol **79 **with Ac_2_O followed by the base catalyzed elimination of acetic acid [81].

### 4.10. Methionine

**Scheme 19:**
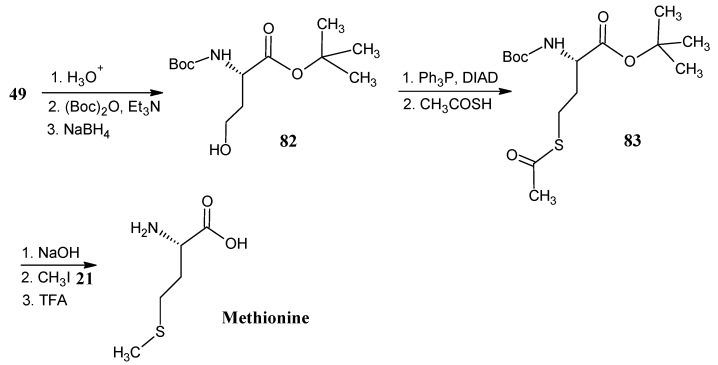
Preparation of methionine from *N*-(diphenylmethylene)aspartic acid 1-*tert*-butyl-4-methyl ester **49**.

Synthetic methods are shown in Scheme 19 for the conversion of *N*-Boc aspartic acid *tert*-butyl ester **49** (Scheme 14) into the alcohol **82**. The reaction steps necessary for this conversion have been described in the Scheme 15 (the conversion of product **51** into product **52**). Mitsonubu reaction with thioacetic acid and DIAD afforded the *N*-Boc protected thioacetate derivative **83**. Treatment of product **83** with a base in the presence of CH_3_I yielded the methylthioether formation to give the required protected methionine that upon deprotection of the amino group with acid afforded methionine [69].

### 4.11. Leucine

**Scheme 20:**
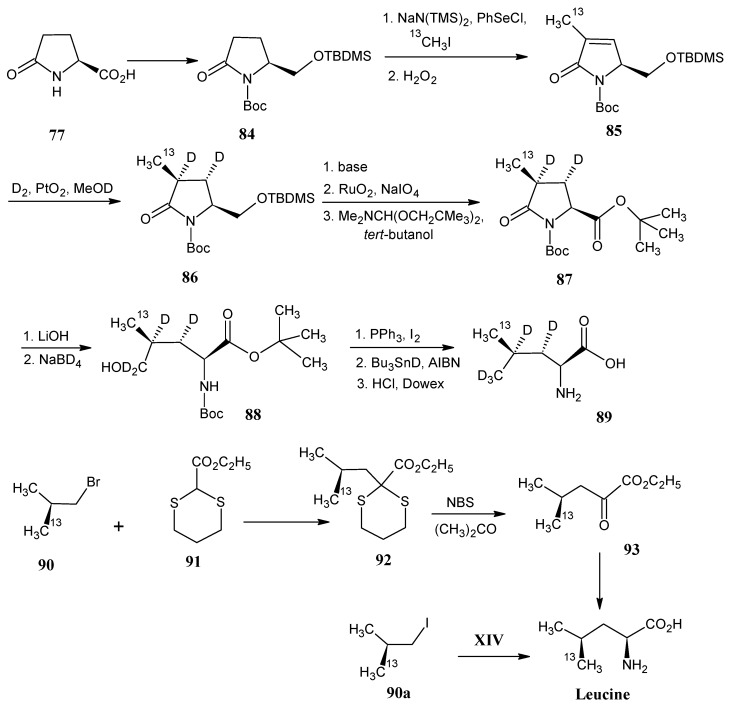
Preparation of enantiomerically pure isotopically labeled leucine starting from pyroglutamic acid **77**.

In Scheme 20 it is depicted that the pyroglutamic acid (**77**, Scheme 18) is converted into the *N*-*tert*-butoxycarbonyl pyroglutamic acid. The carboxylic acid of the product **77** is esterified with ClCO_2_Et/Et_3_N, followed by reduction with NaBH_4_ to obtain the product alcohol which is protectetd with hydroxyl function with *tert*-butyldimethyl silyl chloride (TBDSCl) to afford the product **84**. Strong base induced deprotonation and subsequent treatment with phenyl selenide chloride, followed by treatment with ^13^CH_3_I (**21a**) afforded methylated product that upon reaction with H_2_O_2_ afforded [^13^C]-methylated unsaturated lactam **85**. Treatment of the unsaturated lactam **85** with D_2_ in the presence of catalyst PtO_2_ resulted in the product **86** with stereospecific introduction of deuterium at positions 3 and 4. After removal of the silyl protecting group of the lactam **86** followed by oxidation of the resulting primary alcohol group with RuO_2_/NaIO_4_ the carboxyl function is introduced in the molecule. Treatment with dimethylformamide di-*ter*t-butyl acetal [4-^13^C]-methyl-[3,4-D_2_]-pyroglutamic acid *tert*-butyl ester (**87**) is formed. Base catalyzed ring opening of the amide function and NaBH_4_ reduction yielded the alcohol derivative **88** which is finally converted into (2*S*,3*S*,4*S*)-[5-^13^C;3,4,5′,5′,5′,-D_5_]-leucine (**89**) [82,83].

[2-^13^C]-Methyl propylbromide (**90**) has been prepared via the Evans template method. The ratio of distereomers (2*R*):(2*S*) is 13:1. The conversion of propyl bromide **90** into the α-keto ester **93 ** is effected by the reaction with 2-ethoxycarbonyl-1,3-dithiane (**91**) followed by the oxidative hydrolysis of the resulting product **92** with NBS. Upon reductive amination (2*S*,4*R*)-[5-^13^C]-leucine mixed with 7% of (2*S*,4*S*)-[5-^13^C]-leucine is obtained [84]. The conversion of (2*S*)-[^13^C]-1-iodo-2-methyl propane **90a** into (2*S*,4*R*)-[5-^13^C]-leucine with a mixture of diasteromers (4*R*):(4*S*) in the ratio of 8.5:1, has been effected via O’Donnell method [47].

### 4.12. Isoleucine

**Scheme 21:**
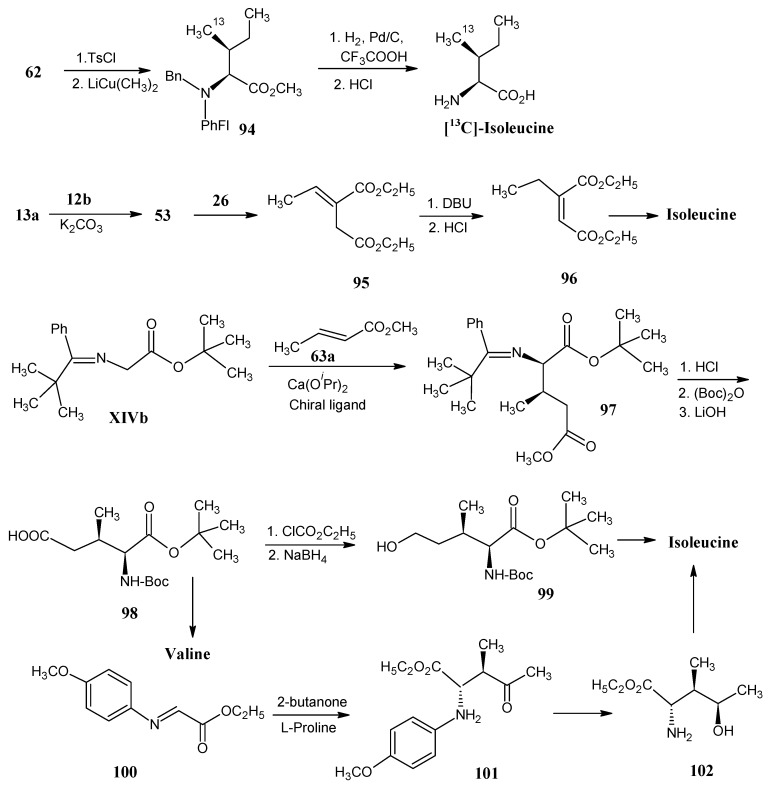
Preparation of isotopically enriched isoleucine.

In Scheme 21 synthetic methods are shown to prepare isoleucine. The hydroxyl function of the valine derivative **62** (Scheme 16) is tosylated and then treated with lithium dimethyl copper to give the protected isoleucine derivative **94** which upon deprotection yielded isoleucine [75]. Acetaldehyde (**26**) reacted with phosphorane **53** to obtain 2-ethylidenebutanedioate (**95**) that upon treatment with DBU the exo-double bond is isomerized to afford 2-ethylbutanedioate 16 it is shown that phosphorane **53** can be obtained by the reaction of an ylide **13a** with ethyl br(**96**). In Scheme omoacetate (**12b**) in the presence of solid K_2_CO_3_. The conversion of 2-ethyl-2-butenedioate (**96**) into the corresponding isoleucine is effected by following a procedure similar to the conversion of the lower homologue 2-methyl fumaric acid (**55**) into valine in Scheme 16 [47].

The *N*-(*tert*-butylphenylmethylene)glycine *tert*-butyl ester **XIVb** reacted with methyl crotonate (**63a**) on a chiral calcium complex (prepared by the reaction of Ca(O*^i^*Pr)_2_ with a chiral catalyst) to give protected (2*R*,3*R*)-3-methyl glutamic acid (**97**) [85]. After saponification of the methyl ester and exchange of the nitrogen protection the N-Boc glutamic acid derivative **98** is obtained which after Barton radical decarboxylation afforded valine [47]. Reaction of glutamic acid derivative **98** with ethyl chloroformate yielded the protected alcohol **99**. Isoleucine is obtained after reduction of the iodine function derived from the alcohol and removal of the *N*-protection group [86].

Treatment of the *N*-*p*-methoxyphenyl protected α-imino ester **100** [accessible by the reaction of glyoxalate (**15a**) and 4-methoxyaniline] with 2-butanone in the presence of L-proline resulted in (2*S*,3*S*)-*N*-*p*-methoxyphenyl protected ester **101** in high yield. Reduction of the ketofunction and deprotection of product **101** afforded (2*S*,3*R*,4*S*)-4-hydroxyisoleucine (**102**). The alkene derivative is obtained upon removal of the hydroxyl function of product **102** and subsequent catalytic reduction of the double bond afforded isoleucine [87].

### 4.13. Lysine

**Scheme 22:**
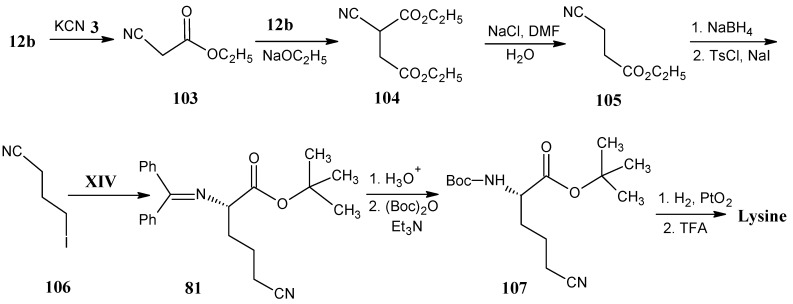
The preparation of stable isotope labeled lysine.

In Scheme 22 it is indicated that ethyl bromoacetate (**12b**) reacted with KCN (**3**) to yield ethyl cyanoacetate (**103**) that is treated with a base and an additional equivalent of ethyl bromoacetate (**12b**) to give ethyl 2-cyanobutanedioate **104**. Diester **104** (in the presence of catalytic amount of NaCl, H_2_O in DMF) is converted into ethyl-4-cyanopropionate (**105**). Selective NaBH_4_ reduction of the ester function into alcohol, subsequent conversion of hydroxyl function into a tosyl group and substitution with iodide ion afforded 4-iodobutyronitrile (**106**) [88].

O’Donnell reaction of 4-iodobutyronitrile (**106**) with **XIV** yielded product **81 **(Scheme 18) that upon deprotection and again the protection with reducing agent stable *N*-protecting Boc group afforded **107**, that upon catalytic reduction of the nitrile function and deprotection afforded lysine [74].

**Scheme 23:**
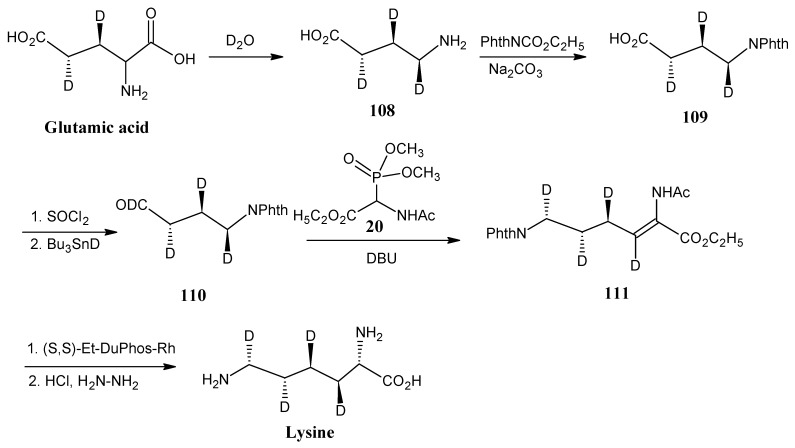
Preparation of deuterated lysine from deuterated glutamic acid.

In Scheme 23 it is indicated that deuterated lysine is prepared from deuterated glutamic acid. The conversion of (2*S*,3*S*,4*R*)-(3,4-^2^H_2_;1,2,3,4,5-^13^C_5_;2-^15^N]-glutamic acid into (2*S*,3*S*,4*R*)-(2,3,4-^2^H_3_;1,2,3,4-^13^C_4_;4-^15^N]-aminobutyric acid (**108**) is achieved by enzymatic decarboxylation with glutamic acid decarboxylase in D_2_O. Subsequent protection of the free amino group into the phthaloyl group afforded the product **109**. The free carboxyl group is converted into acid chloride and reductive deuteration with tributyltin deuteride afforded deuterated aldehyde **110**. The condensation of aldehyde **110** with *N*-acetyl phosphonato glycine ethyl ester (**20**, Scheme 9) in the presence of DBU afforded product **111**. Asymmetric hydrogenation with (+)-1,2-bis[(2S,5S)-2,5-diethylphospholano] benzene-(cyclooctadiene)-rhodium(I)-trifluoro-methanesulfonate [(S,S)-Et-DuPhos-Rh] and deprotection of the amino function by refluxing in HCl, followed by the hydrazine treatment afforded (2*S*,3*R*,4*R*,5*S*,6*R*)-[3,4,5,6-^2^H_4_;1,2,3,4,5,6-^13^C_6_;2,6-^15^N_2_]-lysine [89].

### 4.14. Histidine

**Scheme 24:**
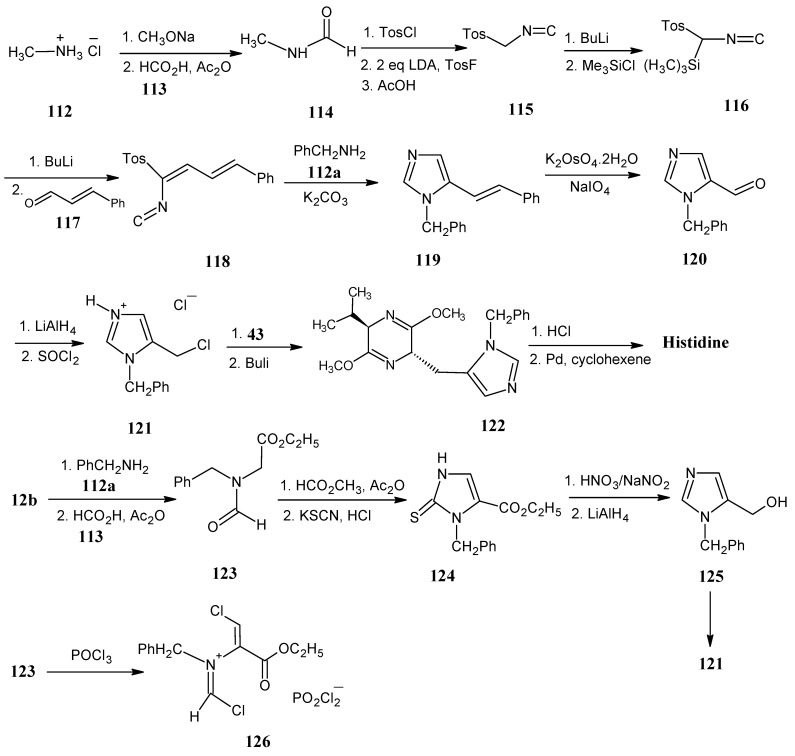
The synthesis of stable isotope enriched histidine from methylammonium chloride (**112**).

In Scheme 24 it is indicated that methylammonium chloride (**112**) after neutralization with sodium methanolate reacted with formic acid (**113**) in acetic anhydride to form *N*-methylformamide (**114**). Upon treatment with tosyl chloride and the base quinoline, methyl isocyanide is formed that is further treated with two equivalents of LDA and then reacted with tosyl fluoride to afford tosylmethyl isocyanide (**115**). Reaction of the product **115** with BuLi and subsequent reaction with trimethylsilyl chloride afforded trimethylsilyl tosylmethyl isocyanide (**116**). The anion of the product **116** is reacted in a Peterson olefination reaction with 3-phenylpropenal (cinnamaldehyde, **117**) to afford the conjugated isocyanide **118**. The isocyanide **118** reacted with benzyl amine (**112a**) and K_2_CO_3_ to form an intermediate imidazolidine ring. With the elimination of *p*-toluenesulfinic acid the imidazole ring is formed to afford the product **119**.

The product **119** is treated with a mixture of potassium osmate (VI) dihydrate (K_2_OsO_4_·2H_2_O) and sodium periodate (NaIO_4_) which cleaved the exo-double bond to afford *N*-benzyl imidazole aldehyde **120**. This molecule can be converted into the (*Z*)-2,3-didehydrohistidine derivative by reaction with the Wittig reagent triethyl phosphonoacetate (not shown in the scheme). The aldehyde function of the product **120** is reduced with LiAlH_4_ and the resulting hydroxyl group is subsequently treated with thionyl chloride to convert it into a chloride. The imidazole group has a pKa of about 7 that afforded the product **121** as a HCl salt.

Originally, product **121** is treated with two eq of the anion **43** of bislactim ether (Schöllköpf method). This led to a loss of one equivalent of anion **43**. The formation of the protected histidine worked well and histidine is isolated after reluxing in HCl and hydrogenation with Pd in cyclohexene [90]. Later the reaction is carried out under O’Donnell conditions. With this method a much milder base at lower pH is used and the formation of the histidine derivatives is smoothly effected [91].

Stable isotope incorporation in 3-phenylpropenal is easily effected by Horner-Wardsworth- Emmons reaction of diethyl phosphonoacetonitrile and benzaldehyde. Subsequent DIBAL reduction converted the nitrile function into the aldehyde function. Diethyl phosphonoacetonitrile can be isotopically labeled at any position via commercially available labeled acetonitrile. ^15^N-Benzylamine has been prepared via the reaction of benzoyl chloride with ^15^NH_3_, subsequent LiAlH_4_ reduction of benzamide afforded benzyl amine. 

Because of the large number of steps involved in the synthesis of the product **119** a new synthetic method is explored with fewer steps. Ethyl bromoacetate (**12b**) is treated with benzyl amine (**112a**) to form ethyl *N*-phenylglycine which upon treatment with formic acid (**113**) in acetic anhydride gave the glycine formamide (**123**). The product **123** underwent a base induced ester condensation with methyl formate to give the enolate of the C-formyl derivative. This molecule reacted with thiocyanate to afford 2-thio imidazolone derivative **124**. Removal of the sulfur is effected by treating it with nitric acid in the presence of NaNO_2_ resulting in the ethyl ester of the protected imidazole compound. LiAlH_4_ reduction gave the imidazole alcohol **125**. This is converted into the imidazole derivative **121** that has been easily converted into histidine in a more efficient way than the first approach [92]. 

It is to be expected that the scheme can be optimized by treating the C-formyl derivative **123**; with POCl_3_ to form the vinyl chloride chloroimidinium salt **126**. Molecules analogous to **126** reacted with NH_4_Cl and Na_2_CO_3_ under substitution of the chloride function to form the benzyl-5-carboethoxyimidazole which upon reaction with LiAlH_4_ afforded the alcohol **125**.

### 4.15. Arginine

**Scheme 25:**
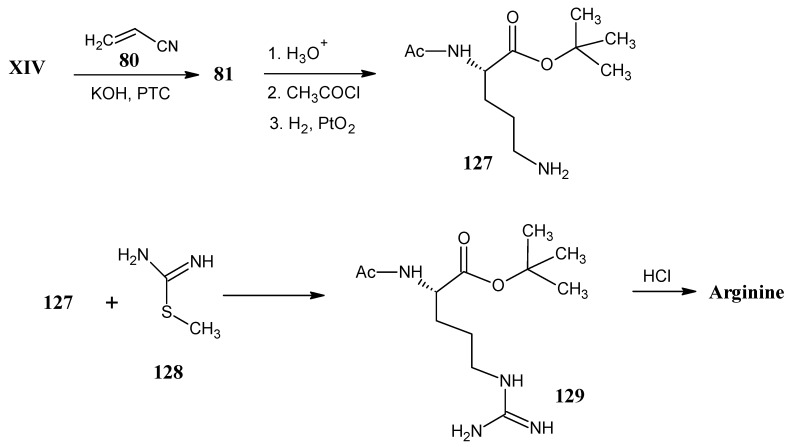
Synthesis of arginine from *N*-acetyl ornithinine *tert*-butyl ester **127**.

In Scheme 25 it is indicated that the reaction of *N*-protected glycine *tert*-butyl ester **XIV** reacted with acrylonitrile (**80**) to afford the nitrile derivative **81** (Scheme 22) that upon *N*-deprotection with acid and subsequent *N*-protection with acetyl chloride afforded the *N*-acetyl protected nitrile. The nitrile derivative is reduced by H_2_ in the presence of PtO_2_ to afford the *N*-protected L-ornithine **127** [74].

The *N*-protected arginine *tert*-butyl ester **129** is obtained by the reaction of *N*-acetyl ornithine *tert*-butyl ester (**127**) with the thiourea derivative (methyl carbamodithioate) **128** [93]. Methyl carbamodithioate (**128**) can be obtained in any stable isotope enriched form by the reaction of NH_4_Cl **1** with potassium thiocyanate (KSCN) followed by the *S*-methylation of thiourea with CH_3_I.

### 4.16. Phenylalanine

**Scheme 26:**
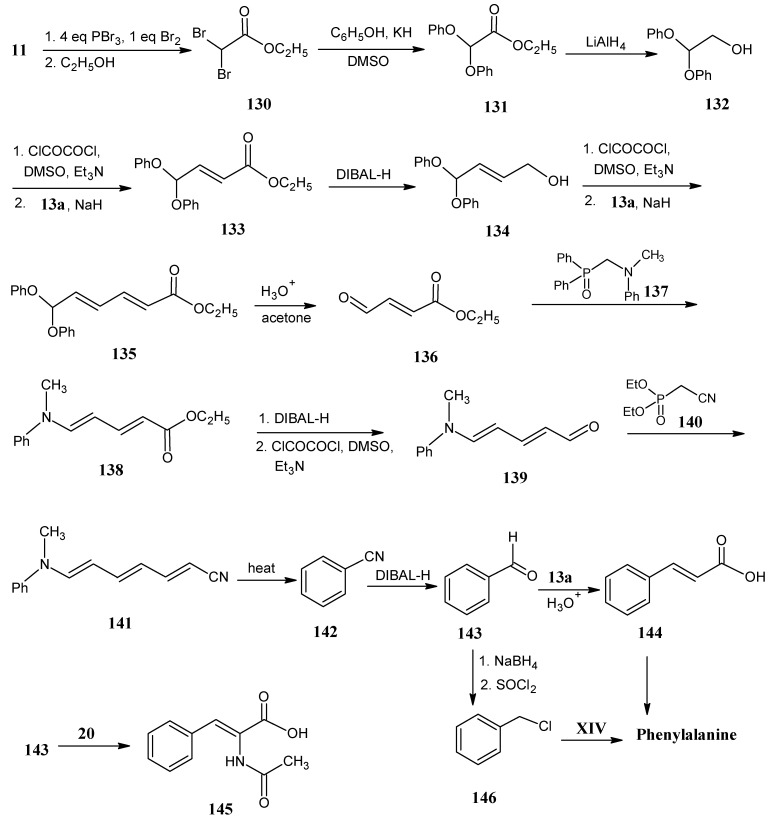
Preparation of stable isotope enriched phenylalanine.

The most difficult part in the preparation of L-phenylalanine is the development of a synthetic scheme that suited for all possible combinations of ^2^H, ^13^C incorporation in the benzene ring. In Scheme 26 a synthetic method is depicted that allows the isotopic enrichment in the benzene ring of phenylalanine.

Acetic acid (**11**) is treated with a four-fold excess of PBr_3_ and one equivalent of Br_2_. This afforded 2,2-dibromoacetic acid that reacted with ethanol to give ethyl 2,2-dibromoacetate (**130**). Upon treatment with phenolate ion the bromine groups are substituted by phenoxy groups to afford the product **131**. 2,2-Diphenoxy ethanol (**132**) is achieved by the LiAlH_4_ reduction of the ester function of the product **131**. Subsequently Swern oxidation of the product **132** with oxalyl chloride in DMSO and triethyl amine afforded 2,2-diphenoxy aldehyde that upon reaction with the anion of ethyl diethylphosphonoacetate **13a** afforded ethyl 4,4-diphenoxy-2-butenoate (**133**) via a Horner-Wadsworth-Emmons reaction. 

Repeating the same sequence for the conversion of ester into alcohol followed by Swern oxidation of alcohol into aldehyde and final Wittig reaction with the ylide **13a** afforded the unsaturated ester **135**. Deprotection of the phenoxy groups in the product **135** afforded the aldehyde ester **136** *in situ* followed by subsequent Horner-Wadsworth-Emmons reaction with the anion of *N*-[(diphenylphosphoryl)methyl]-*N*-methylaniline (**137**) to afford ethyl 5-[methyl(phenyl)amino] penta-2,4-dienoate (**138**). The product **137** is easily obtainable via the the Mannich reaction between *N*-methylaniline and HCHO (**2**) in ethanol, and the resulting intermediate aminal reacted with chlorodiphenylphosphine via an Arbuzov type reaction.

Repeating the reduction of ester group in the product **138**, Swern oxidation of corresponding alcohol afforded penta-2,4-diene-1-al (**139**). Horner-Wadsworth-Emmons reaction with diethyl phosphonoacetonitrile (**140**) gave 1,6-disubstitued hexatriene system **141**. Heating product **141** led to cyclization with the expulsion of *N*-methyl aniline yielding benzonitrile (**142**). In this scheme the building blocks have been used that are easily available in all possible stable isotope enriched forms. The final product benzonitrile (**142**) is therefore now accessible in all possible isotopomeric forms [49].

DIBAL-H reduction of benzonitrile (**142**) afforded benzaldehyde (**143**) that upon Horner-Wadsworth-Emmons coupling with **13a** and subsequent saponification afforded cinnamic acid **144**. Cinnamic acid (**144**) has been enzymatically converted in the presence of NH_3_ into L-phenylalanine [36]. 

Another route is the Wittig reaction of the aldehyde **143** with ethyl ester of *N*-acetyl-2-dimethyl phosphonato glycine (**20**, Scheme 9) to afford 2,3-didehydrophenylalanine (**145**) that has been converted into phenylalanine by asymmetric hydrogenation. An alternative method is the reduction of benzaldehyde (**143**) with NaBH_4_ to obtain benzyl alcohol that is treated with thionyl chloride to obtain benzyl chloride (**146**). This has been reacted with the N-protected glycine **XIV** under O’Donnell conditions to obtain protected phenylalanine [47].

### 4.17. Tyrosine

**Scheme 27:**
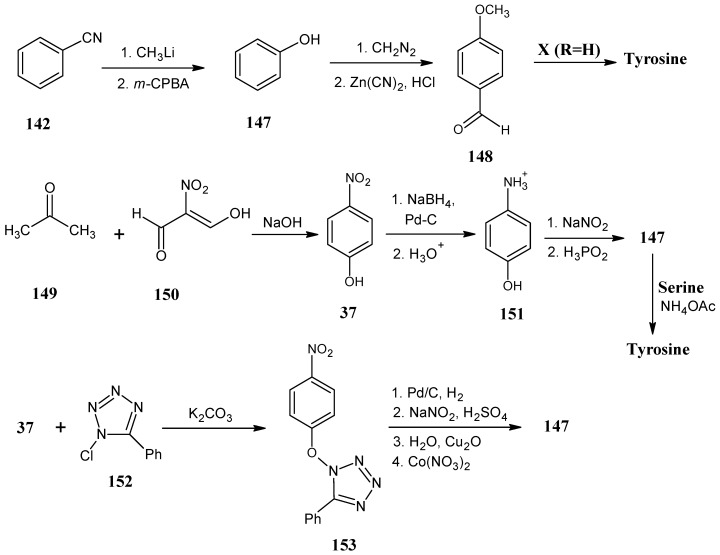
Conversion of benzonitrile (**142**) into tyrosine.

In Scheme 27 the synthetic route for the preparation of tyrosine starting from benzonitrile (**142**) is shown. The compound **142** is treated with methyllithium, followed by acid catalyzed hydrolysis to obtain acetophenone that upon reaction with *m*-chloroperbenzoic acid in water afforded the product phenyl acetate via Bayer-Villiger oxidation. Phenol (**147**) is obtained by hydrolysis of phenyl acetate [49]. Phenol **147** underwent an enzyme catalyzed reaction with serine to give a high yield of tyrosine [36,94]. 

An alternative route is the conversion of phenol (**147**) into anisole by the reaction with diazomethane. A subsequent Gatterman synthesis with Zn(CN)_2_ in the presence of HCl afforded almost quantitatively *p*-methoxybenzaldehyde (**148**) [95]. Condensation of the aldehyde **148** with oxazol-5-(4*H*)-one **X** (R = H) (Scheme 8) and subsequent ring opening afforded 2,3-didehydrotyrosine that upon asymmetric catalytic hydrogenation gave the methyl ether of tyrosine. Final step is the HBr induced removal of the ether function to obtain tyrosine [96].

The schemes discussed so far that allow isotopic enrichment in tyrosine are rather lengthy. For a limited number of ^13^C isotopes in the aromatic ring the reactions in lower line in Scheme 27 have been described. The condensation of acetone (**149**) with 2-nitromalonaldehyde (**150**) under basic conditions afforded *p*-nitrophenol (**37**) in a good yield. Reduction with NaBH_4_ and hydrolysis of *p*-nitrophenol (**37**) yielded aminophenol (**151**). Diazotization with sodium nitrite and reduction of the diazonium ion with hypophosphite resulted in phenol (**147**). Phenol (**147**) is treated with serine in the presence of the enzyme to afford a high yield of tyrosine [94]. Using [1,2,3-^13^C_3_]-labeled acetone (**149**) the ^13^C isotopes are introduced at the carbon positions 1, 2, 6 of *p*-nitrophenol (**37**). In this way tyrosine with ^13^C at positions 3′, 4′ and 5′ has been produced [94]. 

Treatment of the product **37** in the presence of 5-chloro-1-phenyltetrazole (**152**) with potassium carbonate gave the product ether **153**. Hydrogenolysis of the product **153** cleaved the ether bond and simultaneously reduced the nitro function to an amine resulting in aniline which upon diazotization and hydrolysis in water in the presence of Cu_2_O/Co(NO_3_)_2_ afforded phenol (**147**). It is possible to obtain phenol (**147**) enriched with ^13^C isotopes at positions 3′, 4′ and 5′ using this method. The protons ortho to the phenolic hydroxyl function can easily be exchanged for deuterons [97].The preparation of ^17^O and ^18^O nitrophenol (**37**) has been discussed in Scheme 11 [94,97].

The reactions discussed in Scheme 27 afford tyrosine with ^17^O or ^18^O in the phenolic OH group if necessary also in combinations with isotope incorporation in the aliphatic side chain. Schemes that allow to ^17^O or ^18^O incorporation with stable isotope incorporation in the aromatic ring have not been reported. Deuteration at positions 3′ and 5′ in the ring is easily achieved by acid catalyzed deuterium exchange under these conditions without ^17^O or ^18^O exchange [98].

### 4.18. Tryptophan

**Scheme 28:**
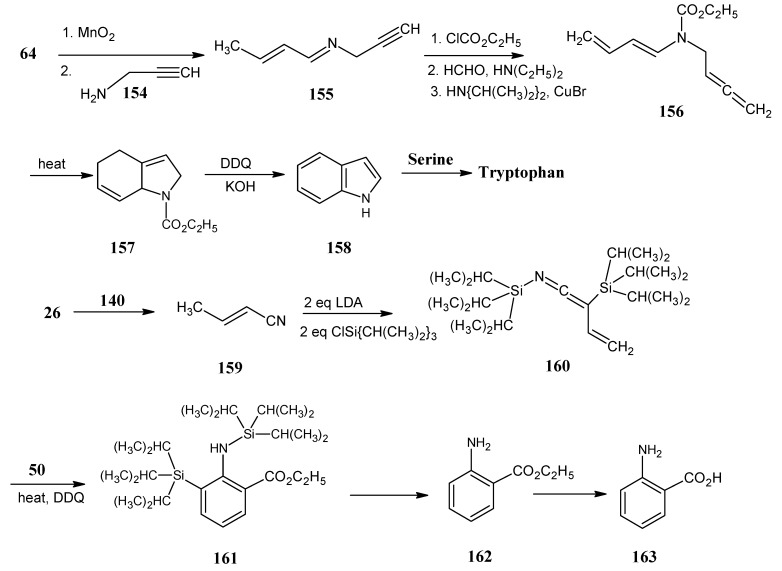
Access to isotope enrichment in indole and side chain of indole at any position and combinations of positions. The preparation of anthranilic acid enriched at any position and combinations of positions with stable isotopes.

In Scheme 28 it is indicated that crotyl alcohol **64** (Scheme 17) is converted into crotonaldehyde after MnO_2_ oxidation and treated with propargyl amine **154** to form the imine **155**.

Reaction of product **154** with ethyl chloroformate converted it into the ethyl carbamate derivative **155** that is treated with HCHO (**2**) in the presence of catalytic amounts of CuBr and diisopropyl amine, the alkyne **155** is converted into the alkene **156**. Product **156** is converted into the tetrahydroindole ester **157** by heating at 160 °C. This molecule is oxidized with two equivalents of dichlorodicyanoquinone (DDQ) to afford the indole ester followed by a base catalyzed saponification to obtain indole **158**. This synthetic method allows for the introduction of isotopes ^15^N, ^13^C at positions 4, 5, 6, 7 and 8, and ^2^H at positions 4, 5, 6 and 7.

At this moment no scheme is available to enrich the isotopes in propargyl amine (**154**). Via an *E. coli*mutant indole (**158**) can be reacted with serine to convert it into tryptophan. ^15^*N*-Anthranilic acid (**163**) can be incorporated into tryptophan residues of protein without ^15^N scrambling or isotope dilution [99,100,101].

The synthetic route for the prepration of anthranilic acid (**163**) is shown in the third line in Scheme 28. Acetaldehyde (**26**) is treated with diethyl phosphonoacetonitrile (**140**) in a Horner-Wadsworth-Emmons reaction to obtain crotononitrile (**159**). Upon reaction with two equivalents of LDA and two equivalents of triisopropylsilyl chloride, the bis-(triisopropylsilyl)-imine **160** is obtained, reacting this molecule with ethyl acrylate (**50**) underwent a Diels-Alder reaction to form the dihydroanthranilic ester derivative. Upon treatment with dichlorodicyanobenzoquinone N-silyl substituted anthranilic ester derivative **161** is obtained. Removal of the triisopropylsilyl group to achieve the ester function **162** and subsequent hydrolysis of the ester group afforded the anthranilic acid (**163**), the molecule is now accessible in any stable isotope enrich form [102].

**Scheme 29:**
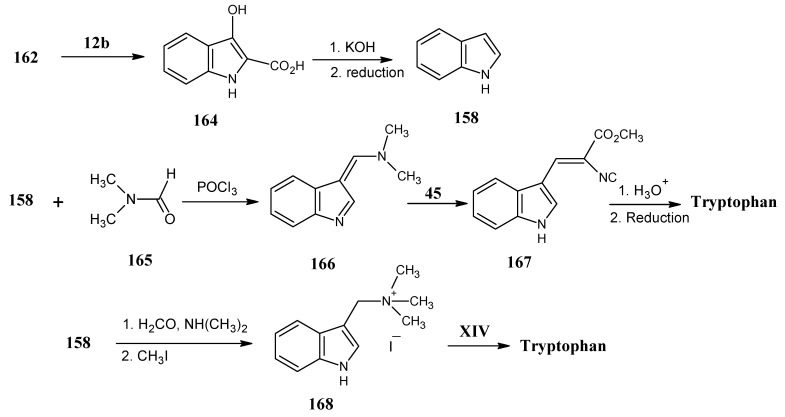
Preparation of indole (**158**) from anthranilic ester **162**.

A synthetic method for the conversion of anthranilic ester (**162**) into indole (**158**) has been depicted In Scheme 29. Anthranilic ester (**162**) is treated with ethyl bromoacetate (**12b**) in the presence of sodium ethanoalate. First, the amino group is alkylated followed by an intramolecular ester condensation to obtain 2-carbethoxy-β-hydroxy indole (**164**) [103]. Treatment of the product **164** with aq. KOH and subsequent acid induced decarboxylation afforded the hydroxy indole which is subsequently reduced to achieve indole (**158)** [104,105].

Indole reacts efficiently with electrophilic reagents. The Vielsmeier-Haack reaction of indole (**158**) with dimethyl formamide **(165**) afforded the indole derivative **166** that is reacted with methyl isocyanoacetate (**45**, Scheme 13) to obtain the isocyano derivative of indole **167**. Mild acid treatment and catalytic asymmetric reduction with D_2_ gives the access to prepare tryptophan specifically deuterated in the aliphatic side chain [106,107]. It is also possible to obtain tryptophan via Mannich reaction of indole (**158**) [108]. 3-Dimethylamino methyl indole is obtained by the treatment of the indole (**158**) with formaldehyde and dimethylamine, followed by the reaction with CH_3_I to afford trimethylammonium iodide **168**. This molecule is treated with the protected glycine under O’Donnell conditions to yield the protected tryptophan [109].

### 4.19. Pyrrolysine

Pyrrolysine is the 22nd genetically encoded amino acid [2]. It consists of a (4*R*,5*R*)-4-methyl-5-carboxypyrroline ring linked to the ε-nitrogen of L-lysine. The access to any stable isotopologue of lysine has been discussed in the paragraph in lysine (Scheme 22 and Scheme 23).

**Scheme 30:**
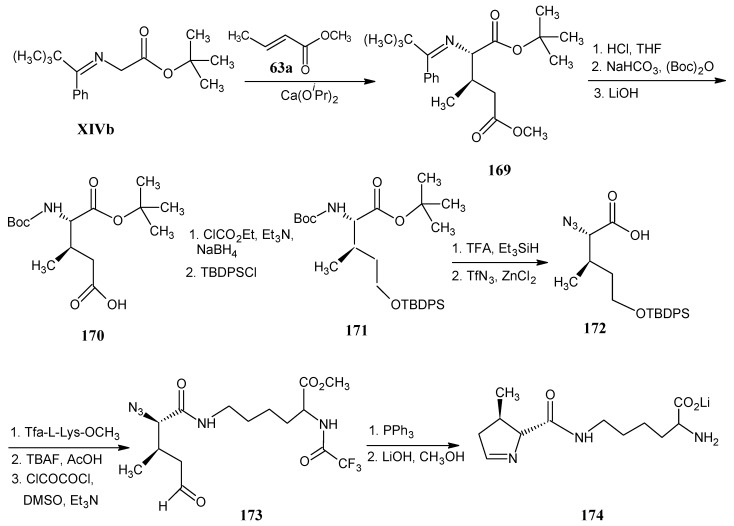
The access to stable isotope labeled (4*R*,5*R*)-4-methyl pyrroline-5-carboxylic acid and its conversion into pyrrolysine.

In Scheme 30 the preparation of the sensitive (4*R*,5*R*)-4-methylpyrroline-5-carboxylic acid is depicted. Base catalyzed 1,4-addition of the anion of *N*-(*tert*-butylphenylmethylidene)glycine *tert*-butyl ester **XIVb** and methyl crotonate (**63a**) in the presence of the optically active catalyst indanol bisoxazoline afforded *N*-(*tert*-butylphenylmethylene)-3-methylglutamic acid *tert*-butyl ester (**169**) with the required (2*R*,3*R*) structure in high enantiomeric excess [86].

The acid derivative **170** is obtained after removal of the amino and carboxylic acid protecting groups. This step is followed by protection of the amino function with a Boc group and reduction of the acid into the alcohol function and protection of the hydroxyl group with *tert*-butyldiphenylsilyl chloride to afford **171**. Hydrolysis of the *tert*-butyl ester and removal of the *N*-Boc protection is achieved by the treatment of the product **171** with trifluoroacetic acid. Treatment of this free amine with triflyl azide under diazo transfer conditions afforded the azide **172**. The azide function is reacted with *N*-trifluoroacetamidyl lysine *O*-methyl ether that reacted with the free ε-amino group of the protected lysine to give an amide bond. Removal of the alcohol protection and subsequent Swern oxidation of the hydroxyl group led to the azide aldehyde derivative **173**. Staudinger reduction of **173** with triphenylphosphine and intramolecular Aza-Wittig reaction afforded pyrrololysine with the protection in the lysine side chain which is removed by treatment with LiOH in methanol to obtain lithium salt of pyrrolysine **174**.

## 5. Conclusions

In this paper the known synthetic schemes to access stable isotope enrichment in the genetically encoded amino acids is reported, together with the stable isotope enrichment of the building blocks. These building blocks are synthesized from the commercially available isotopically labeled starting materials. An essential fact in the syntheses of stable isotope enriched amino acids is that depending on the isotopologues and isotopomers of the required amino acid these schemes can be simplified and the number of synthetic steps can be minimized in a rational way.

With the availability of the full set of isotopomers of the proteinogenic amino acids, all peptides and proteins composed of these amino acids can be labeled at any position or combinations of positions. The isotopically enriched amino acids in the protein will greatly facilitate the study of intra-protein distances, torsion, bond angles and aliphatic-aromatic interactions. With the development of new and better synthetic schemes in the near future to enrich proteins with stable isotopes in an efficient way this will be a preeminent technique in the process of translating structural and functional, biological information etc. at the atomic level of the protein coded by genome into spectroscopic information. 
